# Soluble fibrinogen-like protein 2 promotes the growth of hepatocellular carcinoma via attenuating dendritic cell-mediated cytotoxic T cell activity

**DOI:** 10.1186/s13046-019-1326-5

**Published:** 2019-08-13

**Authors:** Muyang Yang, Zhongwei Zhang, Jiajia Chen, Mengying Xu, Jiaquan Huang, Ming Wang, Weina Li, Xiaoyang Wan, Man-Fung Yuen, Xiaoping Luo, Dong Xi, Qin Ning

**Affiliations:** 1 Institute of Infectious Disease, Tongji Hospital of Tongji Medical College, Huazhong University of Science and Technology, Wuhan, 430030 China; 2 Department of Infectious Disease, Tongji Hospital of Tongji Medical College, Huazhong University of Science and Technology, Wuhan, 430030 China; 3 Department of Medicine, the University of Hong Kong, Queen Mary Hospital, Hong Kong, China; 4 Department of Pediatrics, Tongji Hospital of Tongji Medical College, Huazhong University of Science and Technology, Wuhan, China

**Keywords:** Soluble fibrinogen-like protein 2, Hepatocellular carcinoma, Tumor microenvironment, Dendritic cells, Immunosuppression

## Abstract

**Background:**

Soluble fibrinogen-like protein 2 (sFGL2), a secretory protein expressed by regulatory T cells (Tregs) with immunosuppressive activity, is highly expressed in both the peripheral blood and tumor tissue of patients with hepatocellular carcinoma (HCC); however, sFGL2 function in HCC remains largely unknown. Here, we elucidated the potential role of sFGL2 in HCC progression.

**Methods:**

T cells, dendritic cells (DCs), and related cytokines in the tumor microenvironment were comparatively analyzed in BALB/c and C57BL/6 mice bearing transplanted hepatomas harboring *Fgl2-*knockout or receiving sFGL2-antibody treatment. Additionally, the effects of sFGL2 on DCs and T cells were evaluated in vivo and ex vivo.

**Results:**

The growth of both subcutaneously and orthotopically transplanted hepatomas was inhibited in *Fgl2*-knockout mice and those treated with the sFGL2 antibody, respectively, as compared with controls. Moreover, sFGL2 depletion enhanced the proportion and cytotoxicity of cytotoxic T cells, promoted DC maturation, and improved DC activity to proliferate T cells in the tumor microenvironment. Furthermore, we detected lower levels of interleukin (IL)-35 in both types of transplanted hepatomas and higher level of IL-6 in orthotopically transplanted hepatomas following sFGL2 depletion. Mechanistically, we found that sFGL2 impaired bone-marrow-derived DC (BMDCs) function by inhibiting phosphorylation of Akt, nuclear factor-kappaB, cAMP response element binding protein, and p38 and downregulating the expression of major histocompatibility complex II, CD40, CD80, CD86, and CD83 on BMDCs in vitro.

**Conclusions:**

Our data suggest that sFGL2 promotes hepatoma growth by attenuating DC activity and subsequent CD8^+^ T cell cytotoxicity, suggesting sFGL2 as a novel potential therapeutic target for HCC treatment.

**Electronic supplementary material:**

The online version of this article (10.1186/s13046-019-1326-5) contains supplementary material, which is available to authorized users.

## Background

Hepatocellular carcinoma (HCC), the second leading cause of cancerous mortality, is associated with hepatitis B or C virus infection, which otherwise induces immune tolerance [1, 2]. Resistance to HCC treatment is widely attributed to immune-regulatory cells, such as regulatory T cells (Tregs), myeloid-derived suppressor cells (MDSCs), and tumor-associated macrophages (TAMs) in the tumor microenvironment [3]. Interleukin (IL)-10 and transforming growth factor (TGF)-β, immunosuppressive cytokines secreted mainly by Tregs, are commonly detected in the serum of HCC patients in many cases, with both cytokines capable of inhibiting immune surveillance and protecting tumor growth by attenuating T cell activation [4, 5]. Additionally, immune checkpoint proteins, including cytotoxic T-lymphocyte-associated antigen 4 (CTLA-4), programmed cell death 1 (PD-1), and programmed cell death-ligand 1 (PD-L1), are expressed on Tregs, TAMs, MDSCs, and hepatoma cells and inhibit activation of effector immune cells, such as cytotoxic T cells (CTLs) and natural killer (NK) cells, in tumor tissue [6]. Although attempts have been made to treat HCC with immune-checkpoint inhibitors, efficiency varies in individuals, which limits their use [7, 8]. Therefore, it is important to elucidate an expanded spectrum of immune regulators in the tumor microenvironment in order to identify potential therapeutic targets.

Fibrinogen-like protein 2 (FGL2)/fibroleukin is a member of the fibrinogen-related protein superfamily and comprises both membrane and soluble subtypes [9]. Soluble FGL2 (sFGL2) is an immunosuppressive factor that inhibits dendritic cells (DCs) [10] by binding to the FcγRIIB receptor [11]. As an immune regulator, sFGL2 plays a critical role in the immune balance in autoimmune diseases and can restrict the progression of autoimmune glomerulonephritis [12] and T cell-induced colitis [13]. However, in viral hepatitis, sFGL2 attenuates antiviral immunity, leading to poor prognosis, with impaired Treg function and prolonged survival time observed following FGL2 blockage in murine models of viral fulminant hepatitis [14]. Another study showed that sFGL2 depletion inhibits glioma growth and decreases the numbers of MDSCs, alternatively activated macrophages (M2 macrophages), and CD39^+^ Tregs [15]. Additionally, sFGL2 promotes the accumulation of MDSCs via C-X-C motif chemokine ligand 12 and increases the number of activated cancer-associated fibroblasts in a murine model of lung cancer [16]. In an HCC study, levels of serum sFGL2 were reportedly higher in HCC patients [17], and hepatic stellate cells were found to secrete sFGL2 and inhibit the proliferation of CD8^+^ T cells, thereby hindering antitumor immunity [18]. However, the function of sFGL2 in HCC remains largely unknown.

In this study, we established transplanted hepatoma models and investigated the role of sFGL2 in HCC growth. Our data showed that sFGL2 blockage by antibody interference or genetic deletion decreased hepatoma burden by enhancing DC activation and increasing CTL number and cytotoxicity in tumor tissue while having minimal effect on MDSC and M2 macrophage numbers. Mechanistically, we found that sFGL2 hindered the expression of major histocompatibility complex II (MHCII), CD40, CD80, CD86, and CD83 and attenuated the phosphorylation of Akt, nuclear factor-kappaB (NF-κB), cAMP response element binding protein (CREB), and p38 on bone marrow-derived DCs (BMDCs) in vitro, which influenced DC activation.

## Methods

### Animals

Female wide-type (WT) BALB/c and C57BL/6 mice (aged 6–8 weeks) were purchased from Beijing Vital River Laboratory Animal Technology Co., Ltd. (Beijing, China). Syngeneic *Fgl2*^*−/−*^ mice were generated by the Beijing Genomics Institute (Beijing, China). All mice were kept in micro-isolator cages, and the experimental protocols were approved by the Animal Ethics Committee.

### HCC models

BNL 1ME A.7R.1 cells (BNL cells; 8 × 10^6^) and Hepa1–6 cells (8 × 10^6^) were respectively transplanted into the left flank of BALB/c and C57BL/6 mice to create subcutaneous HCC models. BNL cells are a liver epithelial cell line from BALB/c mice that exhibit malignant properties. Hepa1–6 cells are derived from hepatomas from BW7756 mice and arise in C57BL/6 mice. Tumor volumes were measured every 2 days, and 100 μg of the FGL2 antibody or isotype were injected intratumorally twice weekly once the tumor volume reached > 100 mm^3^. In a separate experiment, the same number of liver cancer cells was inoculated into the left flank of WT and syngeneic *Fgl2*^*−/−*^ mice. Additionally, to explore the effect of IL-35 on the hepatoma environment, 100 μg IL-35 antibody or isotype (Abcam, Cambridge, UK) were injected intra-tumorally once weekly when the tumor volume reached > 100 mm^3^. To establish an orthotopically transplanted HCC model, 1 × 10^6^ BNL cells were implanted into the left lateral liver lobes of WT and syngeneic *Fgl2*^*−/−*^ BALB/c mice.

### Anti-FGL2 polyclonal antibody

For treatment of hepatoma-burdened mice, we used a rabbit polyclonal antibody against a partial form of FGL2 (amino acids 338–356) that included the fibrinogen-related domain critical for immunosuppressive function [19]. The preparation, purification, and identification of the antibody were completed by the Proteintech Group Inc. (Rosemont, IL, USA), which also provided the isotype IgG antibody.

### Cell culture

BNL and Hepa1–6 cells were cultured in Dulbecco’s minimum essential medium containing 10% (v/v) fetal bovine serum (Gibco, Gaithersburg, MD, USA) and 100 μg/mL penicillin/streptomycin (Invitrogen, Carlsbad, CA, USA). In the logarithmic growth phase, cells were harvested and implanted into mice subcutaneously or orthotopically.

Magnetic cell isolation and cell separation (MACS; Miltenyi Biotec, Bergisch Gladbach, Germany) was used to isolate CD4^+^CD25^−^ and CD8^+^ T cells from tumor tissue of untreated WT mice. Subsequently, these cells were mixed with DCs from tumors derived from different groups at various proportions. T cells were dyed with 5 μM carboxyfluorescein succinimidyl amino ester (CFSE) prior to 3-day culture in the presence of 200 IU/mL murine IL-2 (PeproTech, Rocky Hill, NJ, USA) in 96-well round-bottomed plates for 72 h. The proliferated T cells were detected according to the percentage of CFSE dilution. For T helper (Th) cell-differentiation analysis, CD4^+^CD25^−^ T cells were mixed with DCs from tumors at a 5:1 ratio and cultured in the presence of 200 IU/mL murine IL-2 in 96-well round-bottomed plates for 6 days. Th1 and Th2 cells and Tregs were measured and respectively characterized as interferon (IFN)-γ^+^, IL-4^+^, and CD25^+^forkhead box P3^+^ (Foxp3^+^) cells among CD4^+^ T cells.

To analyze CD8^+^ T cell cytotoxicity in tumors, 10^4^ ultraviolet inactivated BNL cells were mixed with 10^5^ CD8^+^ T cells for 72 h. Subsequently, the CD8^+^ T cells were mixed with 10^4^ BNL cells in the logarithmic growth phase for 12 h in order to detect BNL cell apoptosis by 7-aminoactinomycin D (7-AAD) staining.

To obtain BMDCs, bone-marrow cells were separated from mouse tibias, and immature BMDCs were harvested after 6 days of culture with 10 ng/mL IL-4 and 10 ng/mL granulocyte macrophage-colony stimulating factor (GM-CSF; PeproTech).

### Cytokine analysis

IL-12p70, IL-35, and TGF-β in the homogenate were detected using enzyme-linked immunosorbent assay (ELISA) kits (Biolegend, San Diego, CA, USA) at an absorbance of 540 nm. Cytometric bead array (CBA; BD Biosciences, San Diego, CA, USA) was used to measure IL-4, IL-6, IL-10, TNF-α, and IFN-γ levels.

### Flow cytometric analysis

Cell phenotype was assessed by flow cytometry (BD LSRFortessa; BD Biosciences) after incubation with the following fluorescein-labeled antibodies: CD45-allophycocyanin (APC)-eFluor780, CD3e-fluorescein isothiocyanate (FITC), CD4-APC, CD8a-phycoerythrin (PE)-cy7, CD25-PE-cy7, Foxp3-PE, IFN-γ-AlexaFluor488, IL-4-PE-cy7, CD107a-PE, granzymeB-peridinin chlorophyll (PerCP)-eFluor710, perforin-FITC, CD11c-PE-cy7, CD80-PerCP-eFluor710, CD83-FITC, B7-H4-PE, and CD31-PE. All antibodies were purchased from eBioscience (San Diego, CA, USA), except CD4-APC, CD8a-PE-cy7, and Foxp3-PE (BD Biosciences). Intracellular antigens were determined after incubation with ionomycin (500 ng/mL; Abcam, Cambridge, UK) and phorbol-12-myristate13-acetate (10 ng/mL; Sigma-Aldrich, St. Louis, MO, USA) for 1 h and monensin (2 μM; eBioscience) for an additional 4 h. Fixation and permeabilization were performed prior to antibody incubation, and data were analyzed using FlowJo software (TreeStar, Ashland, OR, USA).

### Analysis of DC surface markers and cell-signaling pathways in vitro

BMDCs were incubated with 4 μg/mL recombinant murine FGL2 (R&D Systems, Minneapolis, MN, USA) for 16 h and stimulated with 500 ng/mL lipopolysaccharide (LPS; Sigma-Aldrich) for 12 h. BMDCs were then harvested, and the expression of MHCII, CD40, CD80, CD86, and CD83 was assessed by flow cytometry. To analyze cell-signaling pathways, BMDCs were treated with recombinant murine FGL2 (0, 0.5, 1, or 2 μg/mL) for 16 h, followed by 500 ng/mL LPS stimulation for 30 min. Relevant proteins in cells were extracted and their concentrations measured using a standard bicinchoninic acid assay (Pierce Biotechnology, Rockford, IL, USA). After sodium dodecyl sulfate polyacrylamide gel electrophoresis (SDS-PAGE) and transfer to polyvinylidene fluoride membranes for western blot, 5% not-fat milk was used for blocking for 1 h at room temperature. Primary antibodies were incubated with membranes at 4 °C overnight. Following several wash steps with Tris-buffered saline with Tween-20 (TBST), secondary antibodies were incubated for 1 h at room temperature. Proteins were detected using enhanced chemiluminescence reagent (Servicebio, Wuhan, China), and the integrated optical density (IOD) of the proteins was calculated using a Gel-pro analyzer (Media Cybernetics, Rockville, MD, USA). Antibodies against Akt, phosphorylated (p)-Akt, mechanistic target of rapamycin (mTOR), p-mTOR, NF-κB-p65, p-NF-κB-p65, CREB, p-CREB, p38, p-p38, Erk1/2, and p-Erk1/2 were obtained from Cell Signaling Technology (Danvers, MA, USA), and antibodies for glyceraldehyde 3-phosphate dehydrogenase and histone-3 were purchased from Servicebio.

### Statistical analysis

Data were expressed as mean ± SEM unless otherwise specified. Significance between groups of absolute values was determined by Student’s *t* test, and the Mann–Whitney *U* test was used to analyze percentages. The log-rank test was used to compare survival rates between groups. A *P* < 0.05 was considered significant.

## Results

### FGL2-antibody treatment or *Fgl2* knockout inhibits tumor growth in subcutaneously transplanted HCC models

**Fig. 1:**
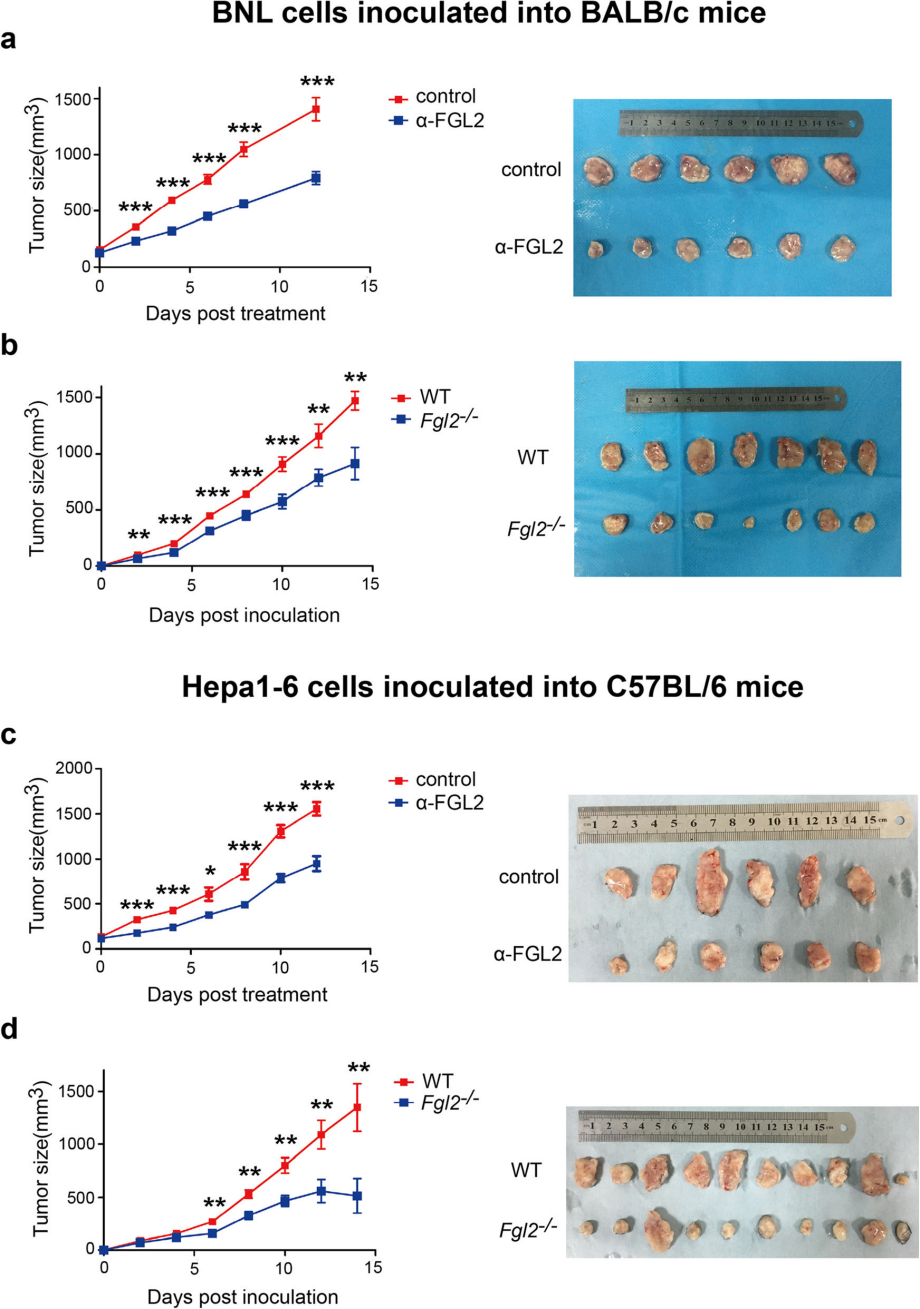
FGL2-antibody treatment or *Fgl2* knockout inhibits the growth of subcutaneously (s.c.) transplanted murine hepatoma**. a** BNL cells (8 × 10^6^) were inoculated s.c. into BALB/c mice, and **c** C57BL/6 mice were injected s.c. with 8 × 10^6^ Hepa1–6 cells. In the anit-FGL2 group, mice were injected with 100 μg anti-FGL2 (ɑ-FGL2) or isotype dissolved in 100 μL phosphate-buffered saline every 3 days after the tumor size exceeded 100 mm^3^. Tumor volumes were measured every 2 days. **b** BNL cells (8 × 10^6^) were inoculated s.c. into *Fgl2*^*−/−*^ or WT BALB/c mice, and **d** *Fgl2*^*−/−*^ and WT C57BL/6 mice were injected s.c. with 8 × 10^6^ Hepa1–6 cells. Tumor volumes were measured every 2 days. *P < 0.05, **P < 0.01, ***P < 0.001 (two-tailed unpaired Student’s *t* test) as compared with the control

To investigate the role of sFGL2 in HCC progression, the growth of BNL or Hepa1–6 cells was evaluated in subcutaneous murine HCC models in *Fgl2*^*−/−*^ mice or treated with the anti-FGL2 antibody in WT mice with a BALB/c or C57BL/6 background. The results showed that FGL2 blockage using the anti-FGL2 antibody (Fig. 1a) or *Fgl2* knockout (Fig. 1b) reduced BNL cell growth in BALB/c mice. Similarly, tumor sizes in C57BL/6 mice bearing Hepa1–6 cells were significantly smaller in groups receiving anti-FGL2 treatment (Fig. 1c) or harboring the *Fgl2* knockout (Fig. 1d) as compared with controls. These data indicated the involvement of sFGL2 in hepatoma progression, and that blockage of sFGL2 represents a potentially novel HCC therapy.

### sFGL2 blockage reduces IL-35 levels, elevates the number of tumor-infiltrated CD8^+^ T cells with enhanced cytotoxicity, and promotes DC maturation in subcutaneously transplanted HCC models

**Fig. 2:**
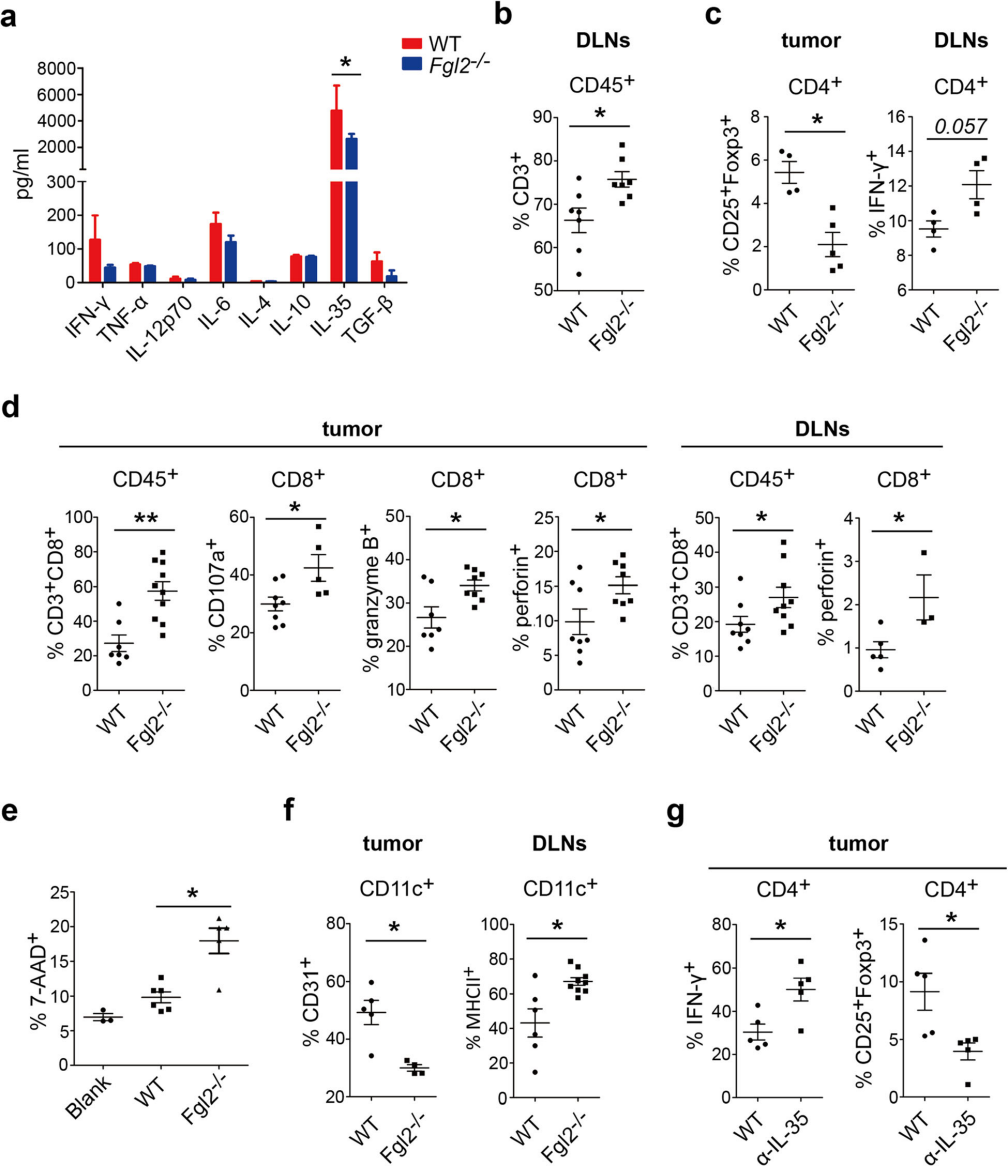
*Fgl2* knockout activates CD8^+^ T cells and DCs in the tumor microenvironment of s.c. transplanted hepatomas. **a** *Fgl2*^*−/−*^ and WT BALB/c mice with s.c. transplanted hepatomas were sacrificed, and cytokines in tumor homogenates were measured by ELISA or CBA at 14-days post-implantation of BNL cells. **b** CD3^+^, **c** CD4^+^, **d** CD8^+^T cells, and **f** DCs in tumors and DLNs were analyzed by flow cytometry. **e** CD8^+^T cells (1 × 10^5^) from s.c. transplanted hepatomas were isolated by magnetic beads and incubated with BNL cells (1 × 10^5^) for 4 days before measuring BNL-cell death percentage. **g** To further insight the influence of diminished IL-35, BNL cells were s.c. transplanted into WT BALB/c mice which were injected with 100 μg IL-35 antibody once weekly when tumor size exceeded 100mm^3^. Immunocytes were measured 14 days after inoculation. Data represent the mean ± SEM. *P < 0.05, **P < 0.01, ***P < 0.001 (**a** two-tailed unpaired Student’s *t* test; **b-f** Mann–Whitney *U* test) compared with negative control

We screened for typical cytokines in tumor homogenates at 2-weeks post-inoculation of BALB/c mice with BNL cells. We found barely detectable levels of sFGL2 in tumor homogenate from *Fgl2*^*−/−*^ mice (Additional file 1: Figure S1a). IL-35 derived from Tregs is a critical immunomodulatory cytokine that inhibits T cell activity and promotes tumor growth. We found significantly lower IL-35 levels in tumor homogenate from *Fgl2*^*−/−*^ mice relative to that from WT mice (2637 ± 383.7 pg/mL vs. 4938 ± 1348 pg/mL; *P* < 0.05) (Fig. 2a). To investigate the immunological mechanism associated with sFGL2-mediated tumor promotion, tumor tissues and draining lymph nodes (DLNs) were dissected and prepared as single-cell suspensions in order to examine immunological features. Measurement of CD3^+^, CD4^+^, and CD8^+^ T cells and DCs by flow cytometric analysis indicated elevated levels of CD3^+^ T cells (Fig. 2b) and Th1 cells (IFN-γ^+^CD4^+^ T cells) (Fig. 2c) in DLNs. Additionally, we observed a 61.3% reduction in Treg percentage among CD4^+^ T cells in BNL-derived tumor tissue from *Fgl2*^*−/−*^ mice (Fig. 2c)*.* Tumor-infiltrated CD8 lymphocytes are the primary sources of antitumor immunity, with their activation characterized by presentation of CD107a and the production of granzyme B and perforin, which kill tumor cells. Here, we found a 2.52-fold increase of CD8^+^ T cells and significantly higher expression of CD107a and production of granzyme B and perforin in tumors from *Fgl2*^*−/−*^ mice, with similar results observed in DLNs (Fig. 2d). To determine the enhanced cytotoxicity of tumor-infiltrated CD8^+^ T cells, the cells were isolated from tumors by MACS and mixed with BNL cells following stimulation with inactivated BNL cells. We observed 1.83-fold increase in the percentage of BNL cells undergoing apoptosis following incubation with CD8^+^ T cells derived from *Fgl2*^*−/−*^ mice, indicating the augmented cytotoxicity of the tumor-infiltrated CD8^+^ T cells (Fig. 2e).

Previous studies demonstrated that sFGL2 hinders DC maturation and function in vitro [10]. To explore whether sFGL2 hampers DCs during HCC progression, we analyzed DCs from the tumors and DLNs of mice subcutaneously transplanted with HCC (Fig. 2f). The proportion of CD31^+^ DCs decreased significantly in the tumor, whereas the number of MHCII^+^ DCs increased in DLNs from *Fgl2*^*−/−*^ mice. Moreover, analysis of the number of MDSCs and M2 macrophages in tumors and DLNs revealed similar levels between *Fgl2*^*−/−*^ and WT mice (Additional file 1: Figure S2a).

We then evaluated the numbers of T cells and DCs in Hepa1–6-derived tumors in C57BL/6 mice, finding a larger number of CD3^+^ T cells in tumors (Additional file 1: Figure S3a) and Th1 cells in tumors and DLNs (Additional file 1: Figure S3b) in *Fgl2*^*−/−*^ mice. Additionally, analysis of CD8^+^ T cells revealed that *Fgl2* knockout elevated the number of tumor-infiltrated CD8^+^ T cells and their production of granzyme B in DLNs (Additional file 1: Figure S3c). Moreover, we observed elevated expression of CD83 on DCs in *Fgl2*^*−/−*^ mice. Furthermore, analysis of the immunological features of T cells and DCs in tumors from BALB/c (Additional file 1: Figure S4) and C57BL/6 (Additional file 1: Figure S5) mice following anti-FGL2 treatment showed similar results. These data suggested that sFGL2 impaired antitumor immunocytes in the hepatoma microenvironment.

Our results showed IL-35 levels was much lower in tumor from *Fgl2*^*−/−*^ mice. To further explore the influence of diminished IL-35, 8 × 10^6^ BNL cells were s.c. injected into BALB/c mice, then 100 μg IL-35 antibody were injected intra-tumorally once weekly when the tumor volume reached > 100 mm^3^. Immunocytes were examined 14 days after tumor inoculation and we found significantly more Th1 cells and fewer Tregs in tumor from anti-IL-35 treatment group compared with control (Fig. 2g).

### sFGL2 blockage promotes DC stimulation of T cells in the tumors of subcutaneously transplanted HCC models

**Fig. 3:**
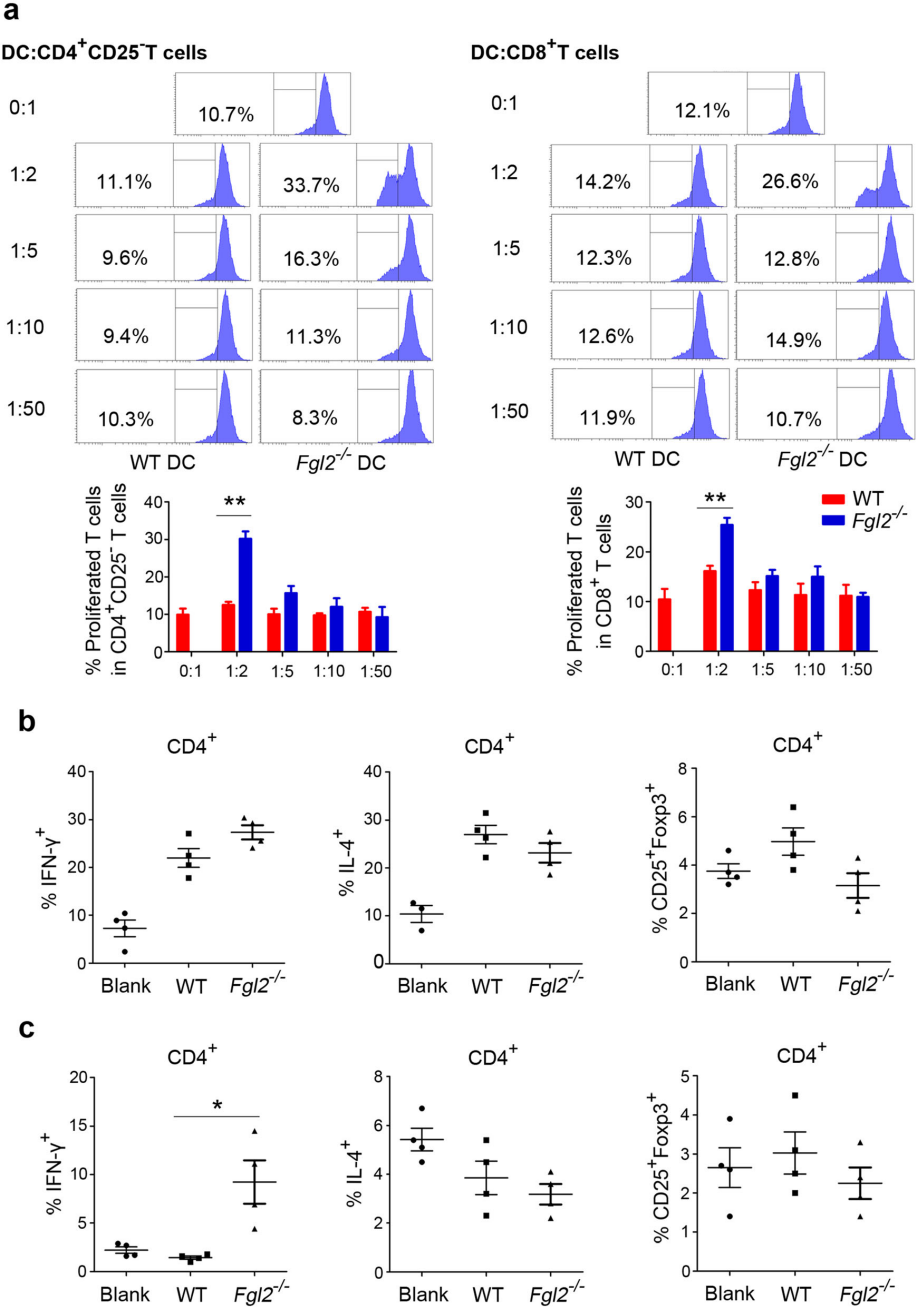
*Fgl2* knockout promotes DC-mediated T cell proliferation in s.c. transplanted hepatomas**.** CD4^+^CD25^−^ and CD8^+^ T cells and DCs were isolated from s.c. transplanted hepatoma tissue from *Fgl2*^*−/−*^ or WT BALB/c mice using magnetic beads. T cells (1 × 10^5^) were dyed with CFSE (5 μM) and mixed with DCs at different ratios (DC:T cells, 1:2, 1:5, 1:10, and 1:50) in the presence of 200 IU/mL murine IL-2 in culture. **a** T cell proliferation was measured after incubation for 72 h in 96-well round-bottomed plates. **b** at a DC:T ratio of 1:2, the percentages of CD4^+^IFN-γ^+^ T cells (Th1), CD4^+^IL-4^+^ T cells (Th2), and CD4^+^CD25^+^Foxp3^+^ T cells (Tregs) among CD4^+^CD25^−^ T cells were quantified after incubation for 6 days. **c** CD4^+^CD25^−^, CD8^+^T cells, and DCs were isolated from s.c. transplanted hepatoma tissue from WT BALB/c mice in the presence or absence of treatment with anti-FGL2. The percentages of Th1 and Th2 cells and Tregs among CD4^+^CD25^−^ T cells were measured as described in (**b**). Data represent the percentage of positive cells (mean ± SEM). *P < 0.05, **P < 0.01 (Mann–Whitney *U* test) as compared with the negative control group

Because variations in the expression of DC-surface molecules do not guarantee changes in DC function, we performed an ex vivo mixed-lymphocyte culture assay. DCs, CD4^+^CD25^−^ effector Th cells, and CD8^+^ T cells from subcutaneously transplanted tumors were isolated by MACS prior to mixing the DCs with different ratios of T cells. The results demonstrated that DCs from mice treated with anti-FGL2 (Additional file 1: Figure S6) or *Fgl2*^*−/−*^ mice (Fig. 3a) displayed an enhance ability to promote both CD4^+^CD25^−^ T and CD8^+^ T cell proliferation at a DC:T cell ratio of 1:2. CD4^+^CD25^−^ effector Th cells and DCs were subsequently mixed and cultured for 6 days, revealing that the percentages of Th2 cells and Tregs among CD4^+^ T cells were not altered by sFGL2 blockage (Fig. 3b and c); however, we observed a significantly greater number of Th1 cells in mice treated with anti-FGL2 as compared with controls (Fig. 3c). These data indicated that sFGL2 depletion enhanced DC function associated with mediation of T cell activation.

### *Fgl2* knockout suppresses the growth of orthotopically transplanted hepatoma and activates tumor-infiltrated CD8 lymphocytes and DCs

**Fig. 4:**
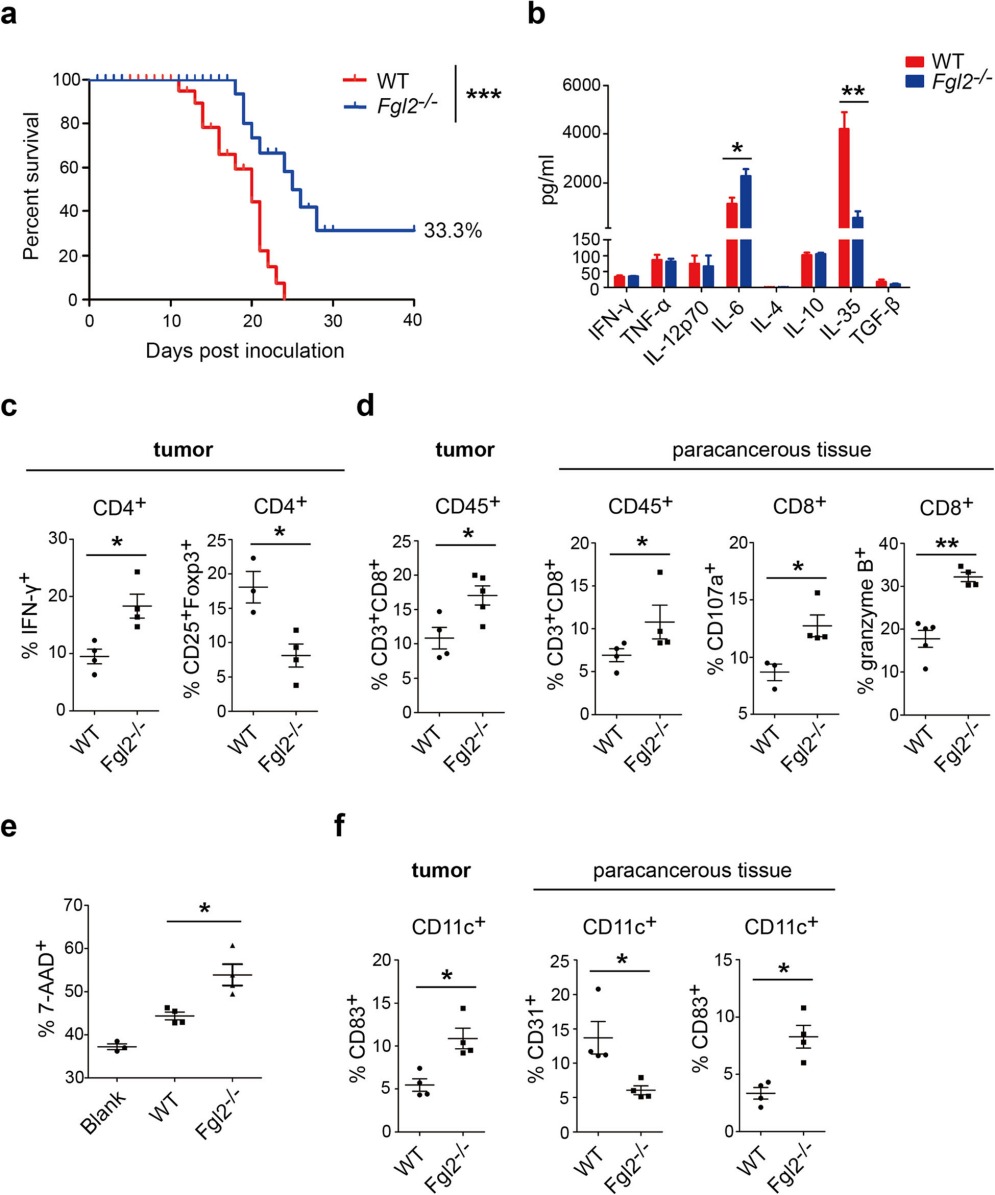
*Fgl2* knockout inhibits hepatoma growth and remodels the tumor microenvironment of an orthotopically transplanted HCC model. **a** BNL cells (1 × 10^6^) were implanted into the left lateral liver lobes of *Fgl2*^*−/−*^ (*n* = 15) or WT (*n* = 15) BALB/c mice, and survival curves were generated. Livers were separated at 14-days post-implantation, and animal-survival rates were calculated using the Kaplan–Meier method. ***P < 0.001 (log-rank test) as compared with the negative control group. **b** Cytokines in liver homogenate were measured by ELISA or CBA. **c** CD4^+^ and **d** CD8^+^ T cells and **f** DCs in tumor and paracancerous tissue were analyzed by flow cytometry. **e** CD8^+^ T cells (1 × 10^5^) from orthotopically transplanted hepatomas were isolated by magnet microbeads and incubated with 1 × 10^4^ BNL cells for 4 days in 96-well round-bottomed plates. CD8^+^ T cells were mixed with BNL cells at a ratio of 10:1 for 12 h before measuring the death percentage of BNL cells. Data represent the mean ± SEM. *P < 0.05, **P < 0.01, ***P < 0.001 (Mann–Whitney *U* test) as compared with the negative control

Because the subcutaneously implanted hepatoma model is not a perfect analogue of HCC in the liver, an orthotopically transplanted HCC model was developed for further verification. Briefly, 1 × 10^6^ BNL cells were implanted into liver lobes of *Fgl2*^*−/−*^ or WT BALB/c mice, after which all 15 WT mice died, and five of the 15 *Fgl2*^*−/−*^ mice survived 40 days after tumor inoculation (Fig. 4a). Tumors were isolated following sacrifice of mice at 14-days post-transplantation from another groups of mice with tumor burden, revealing lower IL-35 (560.7 ± 264.0 pg/mL vs. 4189 ± 694.1 pg/mL; *P* < 0.01) and higher levels of IL-6 levels (2278 ± 288.6 pg/mL vs. 1155 ± 244.2 pg/mL; *P* < 0.05) (Fig. 4b) in tumor homogenate from *Fgl2*^*−/−*^ mice relative to those in WT mice. Additionally, to analyze immunological features, T cells were detected in tumor and paracancerous tissue, revealing twice as many Th1 cells and 50% of Tregs in tumors from *Fgl2*^*−/−*^ mice relative to levels observed in WT mice (Fig. 4c). Moreover, the proportion of CD3^+^CD8^+^ T cells among CD45^+^ cells increased in tumor and paracancerous tissue from *Fgl2*^*−/−*^ mice, accompanied by elevated surface expression of CD107a and production of granzyme B (Fig. 4d), which promoted BNL tumor-cell killing in ex vivo assays (Fig. 4e). Similar to subcutaneous HCC models, we found a higher proportion of mature DCs in tumor and paracancerous tissue in orthotopically transplanted hepatomas in *Fgl2*^*−/−*^ mice along with decreased presentation of CD31 on DCs from both tissues and elevated CD83 expression in tumor tissue (Fig. 4f). However, the numbers of MDSC and M2 macrophages in tumor and paracancerous tissue were not altered by *Fgl2* knockout (Additional file 1: Figure S2b).

### sFGL2 inhibits the expression of MHCII, CD40, CD80, CD86, and CD83 and phosphorylation of Akt and p38 in BMDCs in vitro

**Fig. 5:**
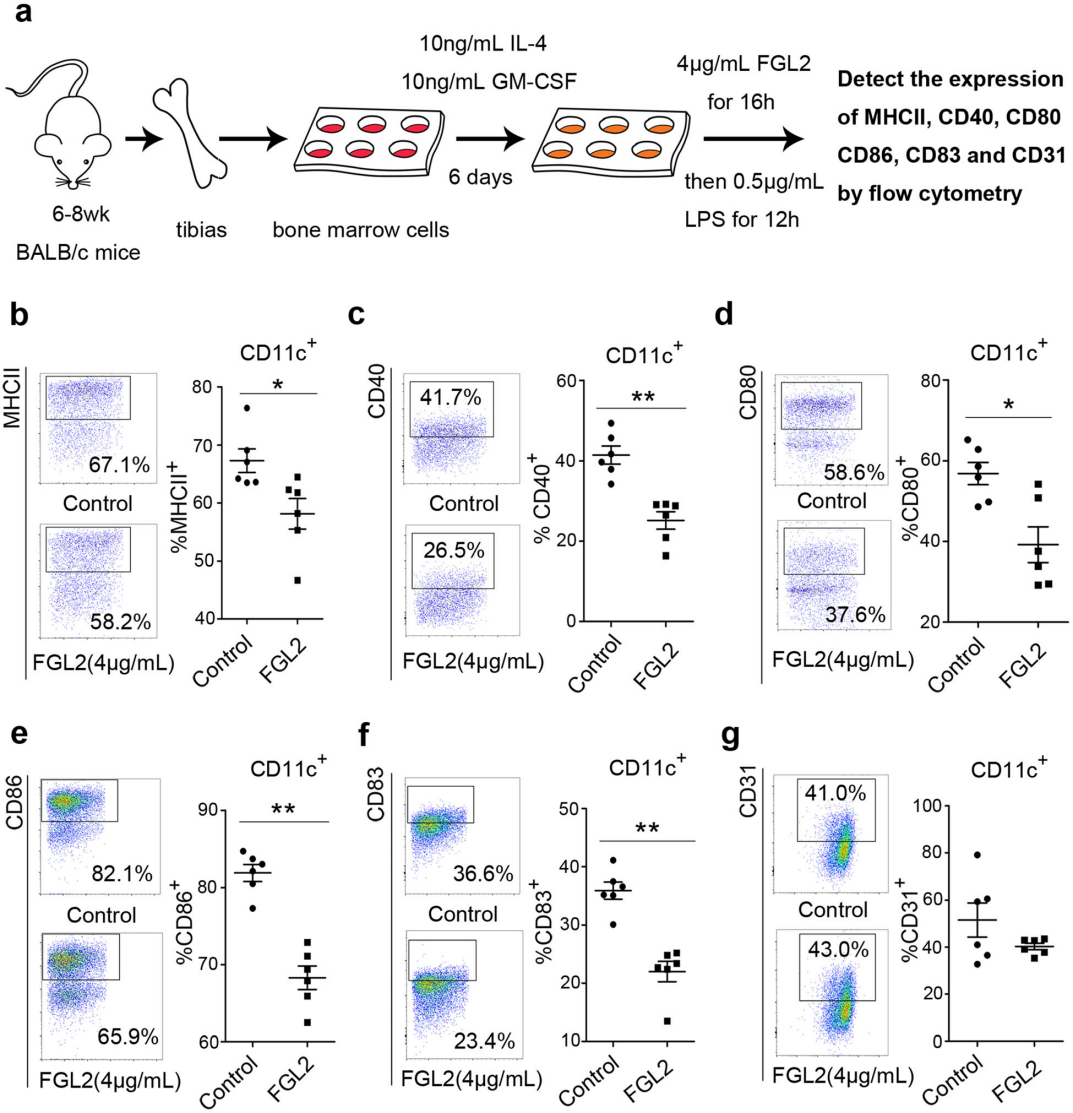
sFGL2 downregulates inflammatory marker expression on DCs**.** Bone-marrow cells were isolated from BALB/c mice and stimulated with 10 ng/mL IL-4 and 10 ng/mL GM-CSF for 6 days in order to generate immature BMDCs, which were then **a** incubated with or without 4 μg/mL of recombinant murine FGL2 for 16 h, followed by 0.5 μg/mL LPS stimulation for 12 h. **b** MHCII, **c** CD40, **d** CD80, **e** CD86, **f** CD83, and **g** CD31 presented on the DC surface were measured by flow cytometry. Data represent the mean ± SEM. *P < 0.05, **P < 0.01 (Mann–Whitney *U* test) and compared with the control

**Fig. 6:**
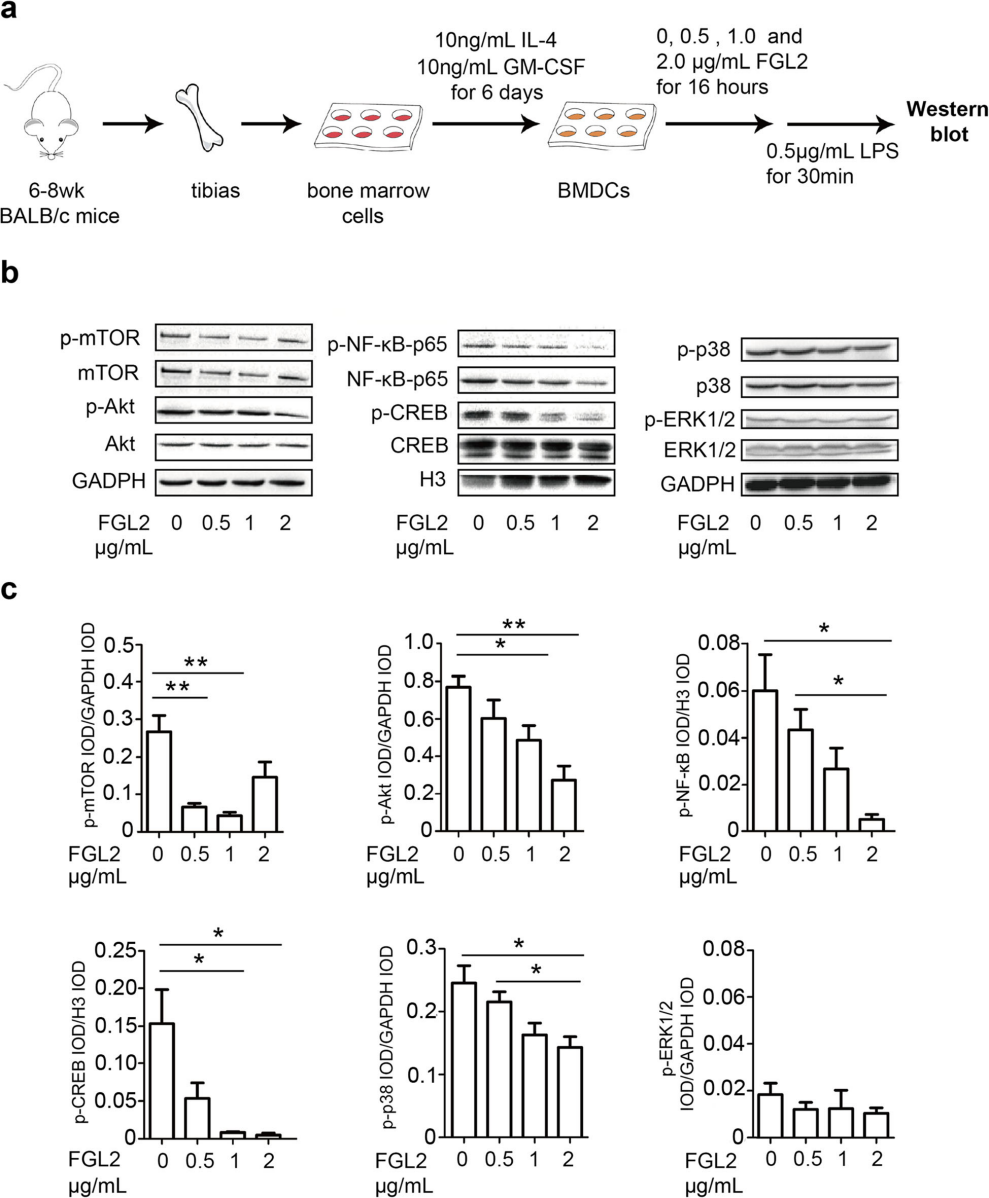
sFGL2 inhibits Akt and p38 phosphorylation and signaling in DCs**.** Bone-marrow cells were isolated from BALB/c mice and stimulated with 10 ng/mL IL-4 and 10 ng/mL GM-CSF for 6 days in order to form immature BMDCs prior to **a** incubation with different concentrations (0, 0.5, 1.0, and 2.0 μg/mL) of recombinant murine FGL2 for 16 h, followed by 0.5 μg/mL LPS stimulation for 30 min. Proteins were detected with anti-mTOR, anti-Akt, anti-NF-κB-p65, anti-CREB, anti-p38, anti-ERK1/2, and their respective phosphorylated forms of the antibodies. **b** A representative result of three independent experiments and **c** the IOD ratio detected protein to that of the internal control are presented as the mean ± SEM. *P < 0.05, **P < 0.01 (Mann–Whitney *U* test) as compared with each other

Given our results showing that sFGL2 blockage leads to DC activation in hepatoma, we then investigated the mechanism associated with sFGL2 abolition of DC activation by culturing immature BMDCs with 4 μg/mL recombinant murine FGL2 for 16 h, followed by stimulation with LPS for 12 h (Fig. 5a). As shown in Fig. 5, sFGL2 significantly reduced the expression of MHCII, CD40, CD80, CD86, and CD83 (Fig. 5b–f). We then determined how sFGL2 influenced Akt, p38, and Erk1/2 phosphorylation in DCs. Akt and its downstream effectors, including NF-κB, CREB, and mTOR, function in diverse cellular processes, including cancer progression and insulin metabolism [20, 21]. p38 mitogen-activated protein kinases (MAPKs) are members of the MAPK family, which is critical to the activation of stress and inflammation [22, 23], and Erk1/2 (p44/p42 MAPK) plays an important role in growth and differentiation [24, 25]. In the present study, our results showed that phosphorylation of Akt, NF-κB p65 subunit, CREB, and p38 was significantly attenuated by higher doses of recombinant murine sFGL2 (Fig. 6). At a dose of 2 μg/mL sFGL2, the IOD of p-mTOR was higher than that observed at 0.5 μg/mL and 1 μg/mL; however p-Erk1/2 levels were not altered by sFGL2. These data indicated that sFGL2 altered Akt, NF-κB, CREB, and p38 phosphorylation and signaling in DCs to possibly change their phenotype and function.

## Discussion

Blockage of immunosuppressive factors is currently regarded as a primary strategy for cancer immune therapy. Tregs, MDSCs, and TAMs are immunosuppressive cells that abate antitumor immunity via the release of immune-regulatory cytokines and presentation of surface molecules, particularly through immune-checkpoint proteins, such as PD-1 and CTLA-4 [26]. Antibodies targeting immune-checkpoint proteins have been demonstrated as effective at controlling the progression of tumor growth in many Phase III clinical trials [27]; however, clinical efficacy is influenced by tumor type, individual response, targets, and drug administration. Moreover, PD-1 and PD-L1 antibodies exhibit limited clinical efficacy in HCC therapy [28], making it necessary to identify new targets.

The immunosuppressive activity of sFGL2 might represent a potentially important mediator of tumor growth. Previous studies report reductions in tumor growth following *Fgl2* knockout in glioma and lung cancer models [15, 16]. In the present study, we reported for the first time that sFGL2 promoted tumor-microenvironment remodeling and HCC progression, and that hepatoma growth was inhibited following blockage of sFGL2 in both subcutaneously and orthotopically transplanted murine models of hepatoma. In glioma and lung tumor models, levels of MDSCs and M2 macrophages decreased in tumors in *Fgl2*-knockout groups, suggesting an sFGL2-mediated mechanism promoting tumor growth through increases in these two subsets [15, 16]. However, in the present study, we found that sFGL2 blockage did not alter the numbers of MDSC and M2 macrophages in HCC models, indicating that sFGL2 might accelerate HCC progression through other pathways.

sFGL2 hinders DC maturation of T cell proliferation in vitro [10]. Therefore, we hypothesized that sFGL2 might promote hepatoma growth by attenuating the number of DCs and T cells, as the main effectors of antitumor immunity. Our results revealed changes in the number, phenotype, and function of DCs and T cells according to sFGL2 status, and that sFGL2 hindered the phosphorylation of mTOR, CREB, Akt, and p38 in BMDCs in vitro. Furthermore, sFGL2 reduced phosphorylated levels of the NF-κB p65 subunit, which subsequently influenced the expression of inflammatory cytokines and surface molecules in DCs. The effect is mediated by sFGL2 binding to FcγRIIB [11] which downregulates the activation of the factors [29]. However, phosphorylation of these factors was unaltered in T cells (Additional file 1: Figure S7), indicating that sFGL2 might not directly modulate T cells. Because changes in Akt and p38 signaling can influence DC maturation and activation [30, 31], we analyzed related surface markers on BMDCs following sFGL2 treatment in vitro, finding that BMDC phenotype was altered by sFGL2 according to decreased levels of MHCII, CD40, CD80, CD86, and CD83 expression. Moreover, in hepatoma-transplanted murine models, sFGL2 blockage promoted MHCII and CD83 expression and downregulated the expression of CD31, which is reportedly an immune-inhibitory marker on the DC surface [32]. These results indicated that sFGL2 blockade promoted DC maturation and inhibited tolerogenic molecule expression on DCs. As the upstream event of adaptive immunity, DC activation can lead to enhanced T cell activation. Our results showed that DCs from the tumor microenvironment in models undergoing sFGL2 blockage upregulated T cell proliferation to a greater degree than that observed in controls. Additionally, DCs from mice treated with the sFGL2 antibody induced higher levels of Th1 differentiation from CD4^+^CD25^−^ T cells. These data confirmed a role for sFGL2 in suppressing DC maturation, resulting in inhibited T cell function.

An increased number of infiltrated CD8^+^ T cells in the HCC microenvironment produced higher levels of perforin and granzyme B following sFGL2 blockade, with this effect augmented by an elevated number of Th1 cells. Here, we observed fewer Tregs in the tumors of sFGL2-depleted mice, suggesting that a deficiency in tolerogenic DCs might have decreased Treg number. However, our data showed that DCs from sFGL2-blocked tumors did not inhibit Treg induction from CD4^+^CD25^−^ cells, indicating that a lower percentage of Tregs in these tumors might not be the results of decreases in the number of DC-induced Tregs. Additionally, we demonstrated that sFGL2 blockage diminished IL-35 levels, which are produced by Tregs. IL-35 plays a vital role of self-tolerance, where IL-35 deficiency promotes autoimmunity in the form of multiple sclerosis, aplastic anemia, allergic rhinitis, and allergic diseases [33]. Moreover, IL-35 promotes tumor growth, with tumor cells overexpressing IL-35 growing faster than controls [34], whereas anti-IL-35 treatment enhances antitumor immunity in vivo [35]. Furthermore, IL-35 in tumor tissue promotes HCC progression and is associated with HCC recurrence [36]. In the present study, we treated BALB/c mice harboring BNL tumors with intratumoral injection of anti-IL-35 once weekly, with the results showing that IL-35 blockage upregulated the number of Th1 cells and diminished the number of Tregs (Fig. 2g). This suggested that IL-35 might be an indicator capable of assessing the tumor microenvironment based on its promotion of tumor growth; therefore, downregulation of IL-35 levels in the hepatoma microenvironment might promote elevations in the number of Th1 cells and decreased Treg infiltration.

## Conclusions

**Fig. 7:**
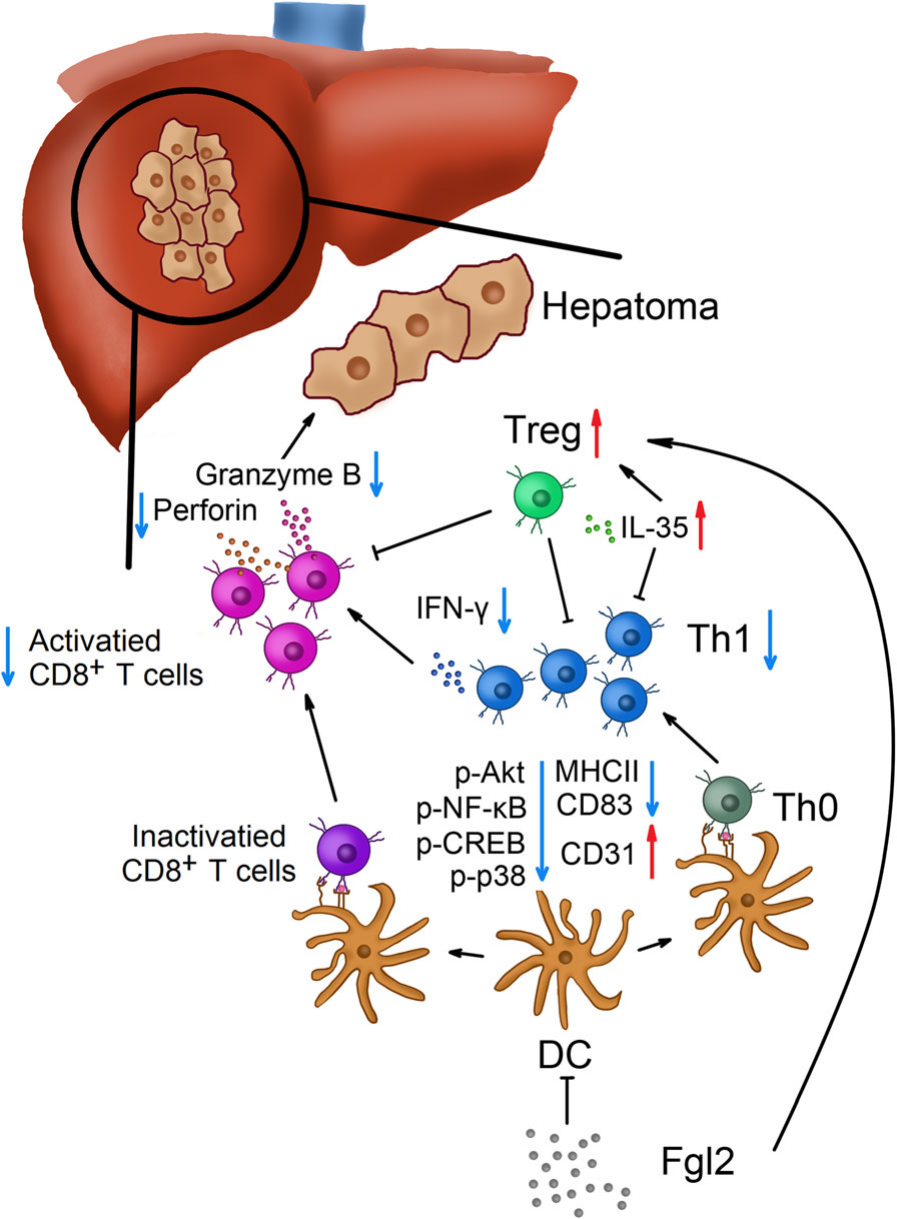
The potential immunological mechanism of sFGL2 in HCC development. sFGL2 attenuates DC activity by inhibiting Akt signaling and induces DCs into a regulatory state. sFGL2 blockage results in attenuated DC expression of CD83 and MHCII and increased expression of CD31, which hinders DC-mediated stimulation of CD8^+^ T cells and Th1 cells. Decreased production of granzyme B and perforin from CD8^+^ T cells exacerbates immunosuppression in the HCC microenvironment. Additionally, the number of Tregs increases along with elevated IL-35 levels. The immunological balance in HCC is thereby disrupted, resulting in dominant immunosuppression and tumor progression

We found that sFGL2 contributes to HCC development, and that its blockage inhibited hepatoma progression. Additionally, sFGL2 inhibited DC activation by abrogating Akt and p38 signaling resulting in the expression of tolerogenic molecules on DCs and impaired inflammatory phenotypes. Moreover, incomplete activation of DCs by sFGL2 resulting in failed priming of CD8^+^ T cells might account for tumor growth. Furthermore, increased Tregs in the tumor microenvironment produced higher levels of IL-35, resulting in attenuated levels of CD8^+^ T cells, Th1 cells, and DCs, which in turn promoted HCC development (Fig. 7). These data support sFGL2 as an important immunomodulatory molecule that might represent a potential therapeutic target for the management of HCC.

## Additional file

Additional file 1: Contains all the supplementary figures and their legends. The titles of the legends are listed below. **Figure S1.** *Fgl2* knockout can diminish significantly sFgl2 level in the hepatoma environment. **Figure S2.** *Fgl2* knockout does not influence the number of MDSCs or M2 macrophages in the hepatoma microenvironment in BALB/c mice. **Figure S3.** *Fgl2* knockout activates CD8^+^ T cells and DC maturation in the tumor microenvironment of s.c. transplanted hepatomas in C57BL/6 mice. **Figure S4.** Anti-FGL2 treatment activates CD8^+^ T cells and DC maturation in the tumor microenvironment of s.c. transplanted hepatomas in BALB/c mice. **Figure S5.** Anti-FGL2 treatment activates CD8^+^ T cells and DC maturation in the tumor microenvironment of s.c. transplanted hepatomas in C57BL/6 mice. **Figure S6.** Anti-FGL2 treatment promotes DC-mediated proliferation of T cells in s.c. transplanted hepatomas in BALB/c mice. **Figure S7.** Akt phosphorylation in T cells is unaltered by sFgl2. (ZIP 2120 kb)

## Data Availability

All data in the study are included in the published article or the supplement files.

## Abbreviations

7-AAD: 7-aminoactinomycin D
APC: Allophycocyanin
BCA: Bicinchoninic acid
BMDCs: Bone marrow-derived dendritic cells
CBA: Cytometric beads array
CFSE: Carboxyfluorescein succinimidyl amino ester
CREB: CAMP response element binding protein
CTLA-4: Cytotoxic T-lymphocyte-associated antigen 4
CTLs: Cytotoxic T lymphocytes
DCs: Dendritic cells
DLNs: Draining lymph nodes
ELISA: Enzyme linked immunosorbent assay
ERK: Extracellular signal-regulated kinase
FGL2: Fibrinogen-like protein 2
FITC: Fluorescein isothiocyanate
Foxp3: Forkhead box P3
GM-CSF: Granulocyte macrophage-colony stimulating factor
HCC: Hepatocellular carcinoma
IFN: Interferon
IL: Interleukin
IOD: Integrated optical density
LPS: Lipopolysaccharide
MACS: Magnetic cell isolation and cell separation
MAPK: Mitogen-activated protein kinase
MDSCs: Myeloid-derived suppressor cells
MHCII: Major histocompatibility complex II
mTOR: Mechanistic target of rapamycin
NF-κB: Nuclear factor-kappaB
NK: Natural killer
PD-1: Programmed cell death 1
PD-L1: Programmed cell death-ligand 1
PE: Phycoerythrin
PerCP: Peridinin chlorophyll
PMA: Phorbol-12-myristate-13-acetate
SDS-PAGE: Sodium dodecyl sulfate polyacrylamide gel electrophoresis
sFGL2: Soluble fibrinogen-like protein 2
TAM: Tumor-associated macrophages
TBST: Tris-buffered saline with Tween-20
TGF: Transforming growth factor
Th1: Type 1 T helper cells
Th2: Type 2 T helper cells
TNF: Tumor necrosis factor
Tregs: Regulatory T cells
WT: Wide-type
