# Supplementary figures and images for: Soluble fibrinogen-like protein 2 promotes the growth of hepatocellular carcinoma via attenuating dendritic cell-mediated cytotoxic T cell activity
Source: J Exp Clin Cancer Res. 2019 Aug 13;38:351. doi: 10.1186/s13046-019-1326-5 (PMC6693134; doi:10.1186/s13046-019-1326-5)

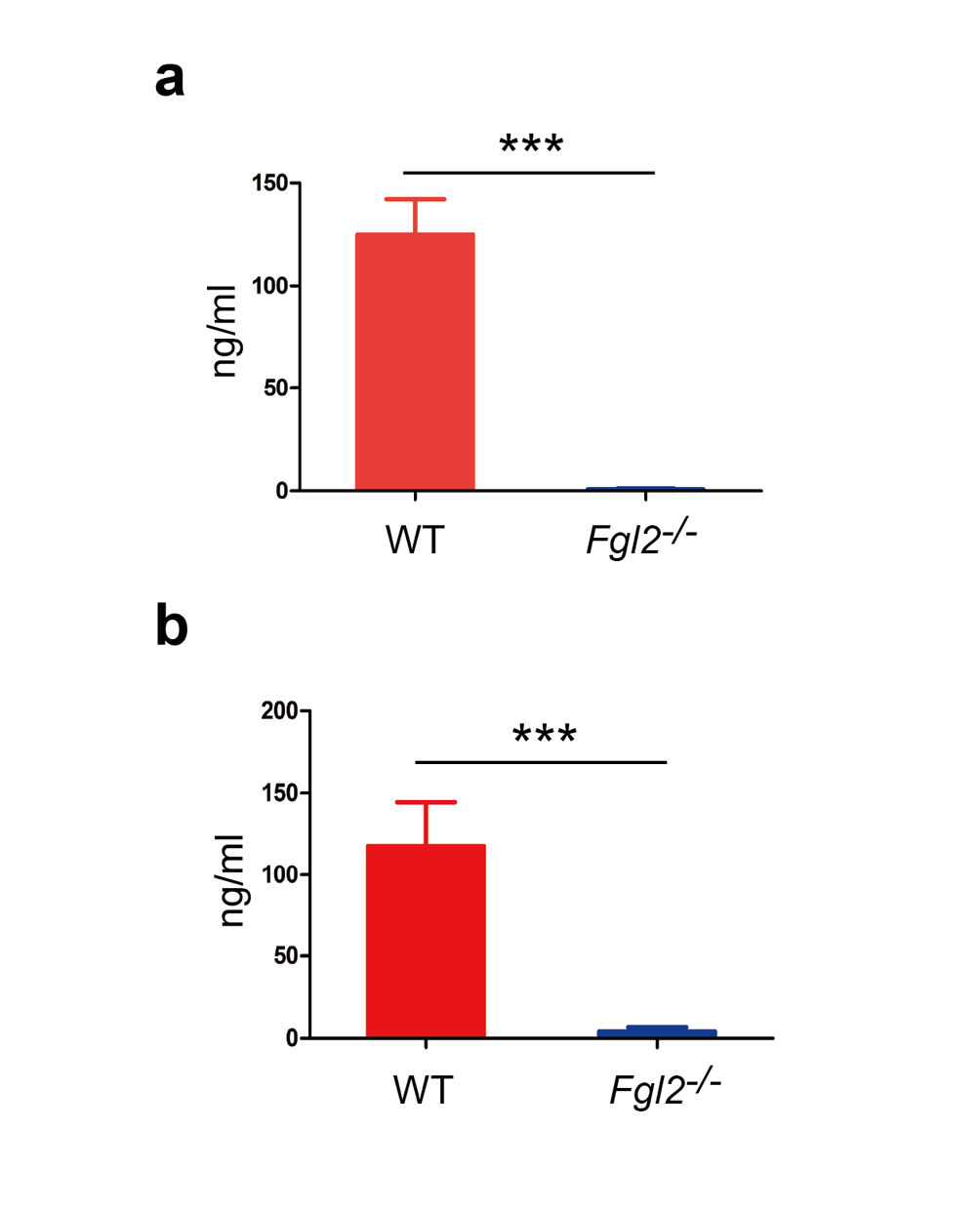

Supplement: Supplementary file 1 — Contains all the supplementary figures and their legends. The titles of the legends are listed below. Figure S1. Fgl2 knockout can diminish significantly sFgl2 level in the hepatoma environment. Figure S2. Fgl2 knockout does not influence the number of MDSCs or M2 macrophages in the hepatoma microenvironment in BALB/c mice. Figure S3. Fgl2 knockout activates CD8+ T cells and DC maturation in the tumor microenvironment of s.c. transplanted hepatomas in C57BL/6 mice. Figure S4. Anti-FGL2 treatment activates CD8+ T cells and DC maturation in the tumor microenvironment of s.c. transplanted hepatomas in BALB/c mice. Figure S5. Anti-FGL2 treatment activates CD8+ T cells and DC maturation in the tumor microenvironment of s.c. transplanted hepatomas in C57BL/6 mice. Figure S6. Anti-FGL2 treatment promotes DC-mediated proliferation of T cells in s.c. transplanted hepatomas in BALB/c mice. Figure S7. Akt phosphorylation in T cells is unaltered by sFgl2. (ZIP 2120 kb) [file 13046_2019_1326_MOESM1_ESM.zip › FigS1.tif]

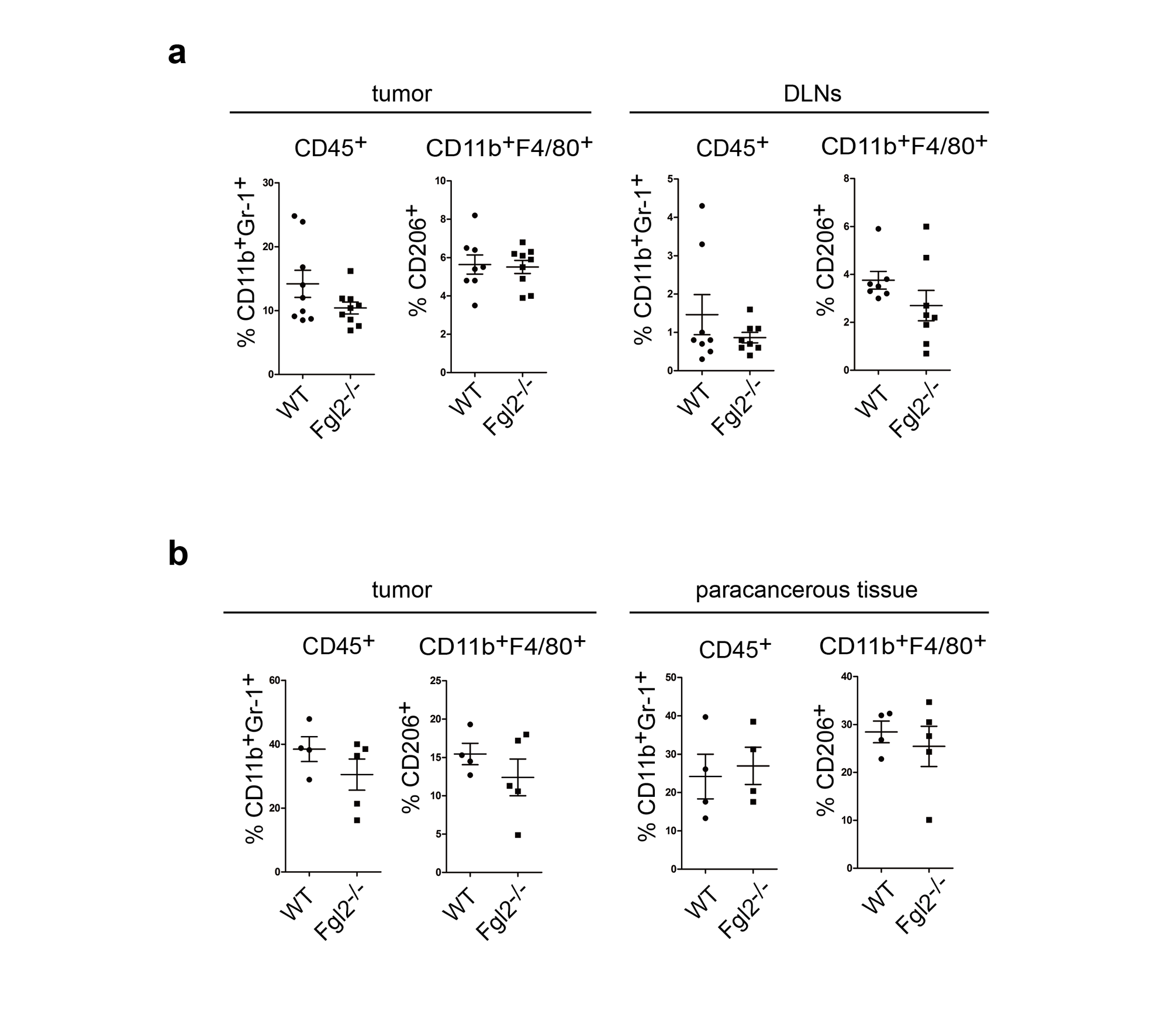

Supplement: Supplementary file 1 — Contains all the supplementary figures and their legends. The titles of the legends are listed below. Figure S1. Fgl2 knockout can diminish significantly sFgl2 level in the hepatoma environment. Figure S2. Fgl2 knockout does not influence the number of MDSCs or M2 macrophages in the hepatoma microenvironment in BALB/c mice. Figure S3. Fgl2 knockout activates CD8+ T cells and DC maturation in the tumor microenvironment of s.c. transplanted hepatomas in C57BL/6 mice. Figure S4. Anti-FGL2 treatment activates CD8+ T cells and DC maturation in the tumor microenvironment of s.c. transplanted hepatomas in BALB/c mice. Figure S5. Anti-FGL2 treatment activates CD8+ T cells and DC maturation in the tumor microenvironment of s.c. transplanted hepatomas in C57BL/6 mice. Figure S6. Anti-FGL2 treatment promotes DC-mediated proliferation of T cells in s.c. transplanted hepatomas in BALB/c mice. Figure S7. Akt phosphorylation in T cells is unaltered by sFgl2. (ZIP 2120 kb) [file 13046_2019_1326_MOESM1_ESM.zip › FigS2.tif]

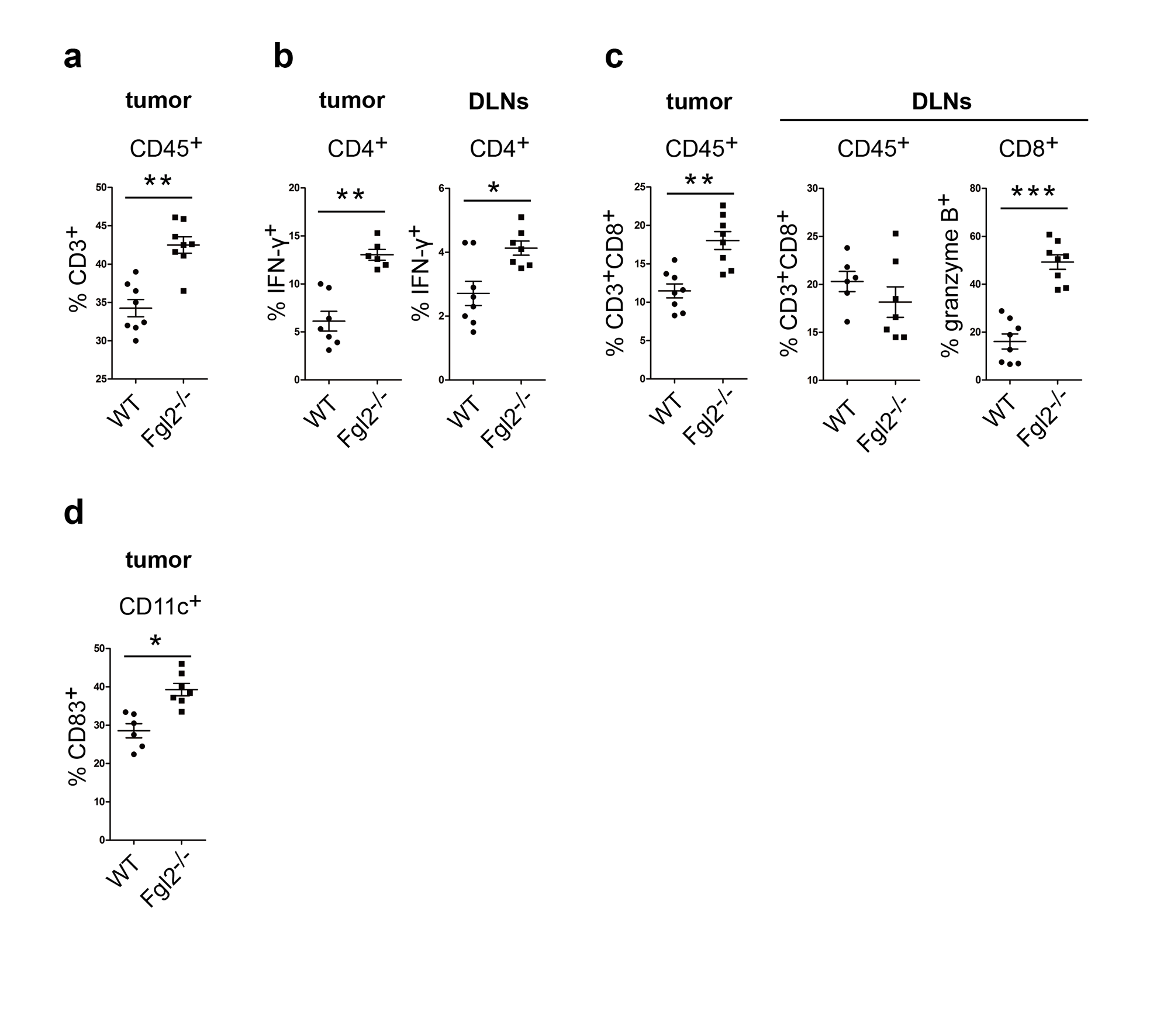

Supplement: Supplementary file 1 — Contains all the supplementary figures and their legends. The titles of the legends are listed below. Figure S1. Fgl2 knockout can diminish significantly sFgl2 level in the hepatoma environment. Figure S2. Fgl2 knockout does not influence the number of MDSCs or M2 macrophages in the hepatoma microenvironment in BALB/c mice. Figure S3. Fgl2 knockout activates CD8+ T cells and DC maturation in the tumor microenvironment of s.c. transplanted hepatomas in C57BL/6 mice. Figure S4. Anti-FGL2 treatment activates CD8+ T cells and DC maturation in the tumor microenvironment of s.c. transplanted hepatomas in BALB/c mice. Figure S5. Anti-FGL2 treatment activates CD8+ T cells and DC maturation in the tumor microenvironment of s.c. transplanted hepatomas in C57BL/6 mice. Figure S6. Anti-FGL2 treatment promotes DC-mediated proliferation of T cells in s.c. transplanted hepatomas in BALB/c mice. Figure S7. Akt phosphorylation in T cells is unaltered by sFgl2. (ZIP 2120 kb) [file 13046_2019_1326_MOESM1_ESM.zip › FigS3.tif]

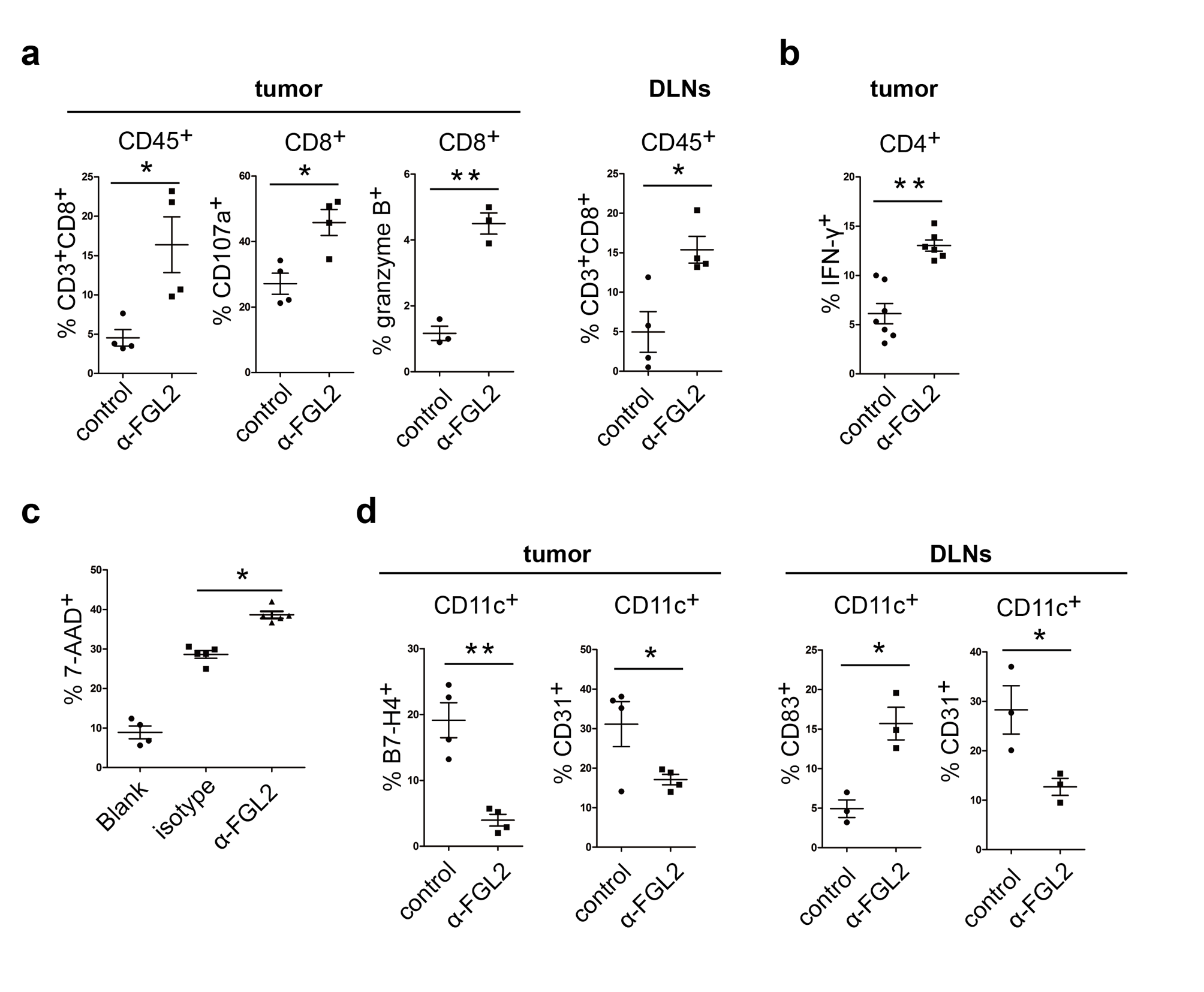

Supplement: Supplementary file 1 — Contains all the supplementary figures and their legends. The titles of the legends are listed below. Figure S1. Fgl2 knockout can diminish significantly sFgl2 level in the hepatoma environment. Figure S2. Fgl2 knockout does not influence the number of MDSCs or M2 macrophages in the hepatoma microenvironment in BALB/c mice. Figure S3. Fgl2 knockout activates CD8+ T cells and DC maturation in the tumor microenvironment of s.c. transplanted hepatomas in C57BL/6 mice. Figure S4. Anti-FGL2 treatment activates CD8+ T cells and DC maturation in the tumor microenvironment of s.c. transplanted hepatomas in BALB/c mice. Figure S5. Anti-FGL2 treatment activates CD8+ T cells and DC maturation in the tumor microenvironment of s.c. transplanted hepatomas in C57BL/6 mice. Figure S6. Anti-FGL2 treatment promotes DC-mediated proliferation of T cells in s.c. transplanted hepatomas in BALB/c mice. Figure S7. Akt phosphorylation in T cells is unaltered by sFgl2. (ZIP 2120 kb) [file 13046_2019_1326_MOESM1_ESM.zip › FigS4.tif]

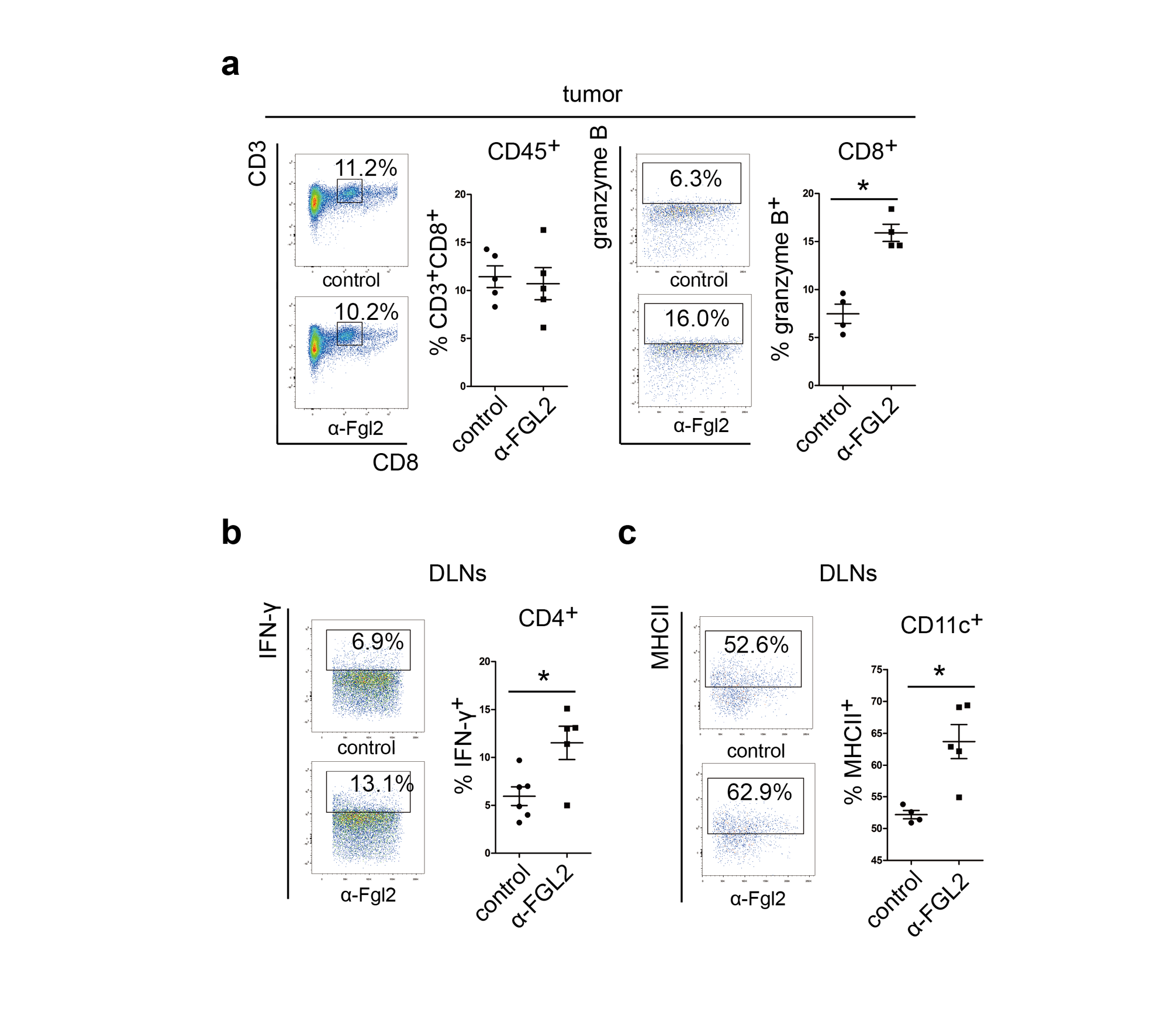

Supplement: Supplementary file 1 — Contains all the supplementary figures and their legends. The titles of the legends are listed below. Figure S1. Fgl2 knockout can diminish significantly sFgl2 level in the hepatoma environment. Figure S2. Fgl2 knockout does not influence the number of MDSCs or M2 macrophages in the hepatoma microenvironment in BALB/c mice. Figure S3. Fgl2 knockout activates CD8+ T cells and DC maturation in the tumor microenvironment of s.c. transplanted hepatomas in C57BL/6 mice. Figure S4. Anti-FGL2 treatment activates CD8+ T cells and DC maturation in the tumor microenvironment of s.c. transplanted hepatomas in BALB/c mice. Figure S5. Anti-FGL2 treatment activates CD8+ T cells and DC maturation in the tumor microenvironment of s.c. transplanted hepatomas in C57BL/6 mice. Figure S6. Anti-FGL2 treatment promotes DC-mediated proliferation of T cells in s.c. transplanted hepatomas in BALB/c mice. Figure S7. Akt phosphorylation in T cells is unaltered by sFgl2. (ZIP 2120 kb) [file 13046_2019_1326_MOESM1_ESM.zip › FigS5.tif]

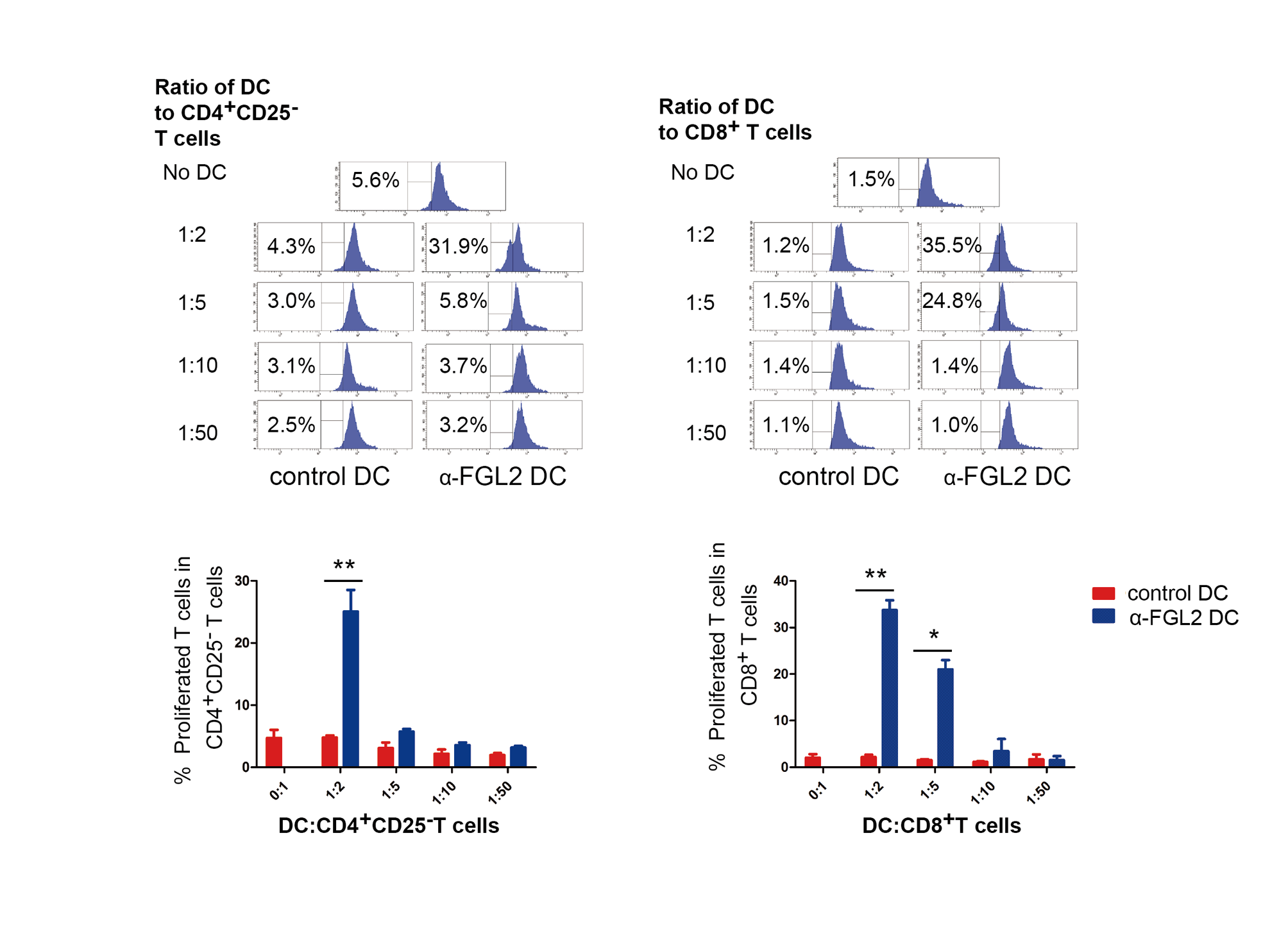

Supplement: Supplementary file 1 — Contains all the supplementary figures and their legends. The titles of the legends are listed below. Figure S1. Fgl2 knockout can diminish significantly sFgl2 level in the hepatoma environment. Figure S2. Fgl2 knockout does not influence the number of MDSCs or M2 macrophages in the hepatoma microenvironment in BALB/c mice. Figure S3. Fgl2 knockout activates CD8+ T cells and DC maturation in the tumor microenvironment of s.c. transplanted hepatomas in C57BL/6 mice. Figure S4. Anti-FGL2 treatment activates CD8+ T cells and DC maturation in the tumor microenvironment of s.c. transplanted hepatomas in BALB/c mice. Figure S5. Anti-FGL2 treatment activates CD8+ T cells and DC maturation in the tumor microenvironment of s.c. transplanted hepatomas in C57BL/6 mice. Figure S6. Anti-FGL2 treatment promotes DC-mediated proliferation of T cells in s.c. transplanted hepatomas in BALB/c mice. Figure S7. Akt phosphorylation in T cells is unaltered by sFgl2. (ZIP 2120 kb) [file 13046_2019_1326_MOESM1_ESM.zip › FigS6.tif]

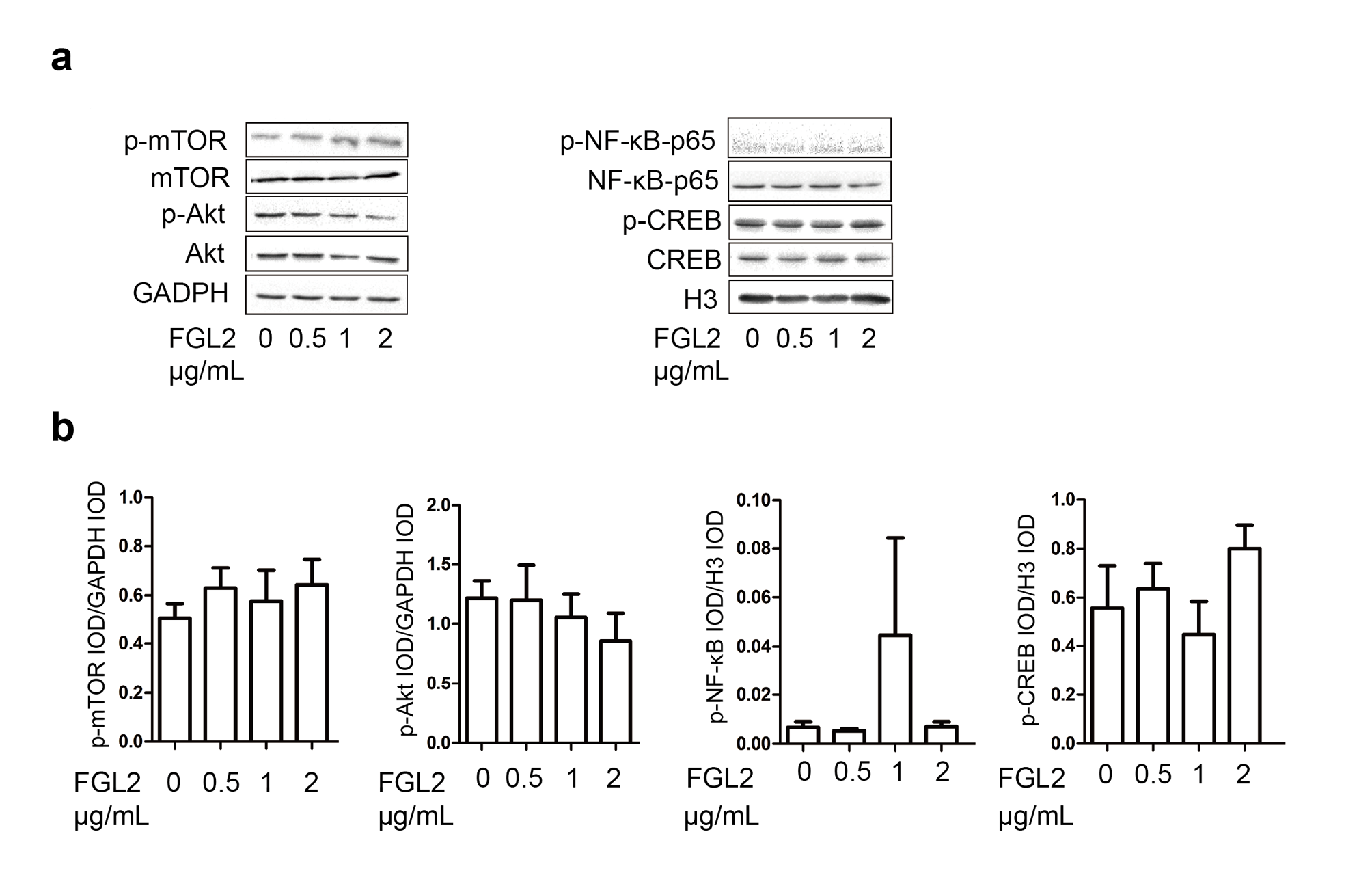

Supplement: Supplementary file 1 — Contains all the supplementary figures and their legends. The titles of the legends are listed below. Figure S1. Fgl2 knockout can diminish significantly sFgl2 level in the hepatoma environment. Figure S2. Fgl2 knockout does not influence the number of MDSCs or M2 macrophages in the hepatoma microenvironment in BALB/c mice. Figure S3. Fgl2 knockout activates CD8+ T cells and DC maturation in the tumor microenvironment of s.c. transplanted hepatomas in C57BL/6 mice. Figure S4. Anti-FGL2 treatment activates CD8+ T cells and DC maturation in the tumor microenvironment of s.c. transplanted hepatomas in BALB/c mice. Figure S5. Anti-FGL2 treatment activates CD8+ T cells and DC maturation in the tumor microenvironment of s.c. transplanted hepatomas in C57BL/6 mice. Figure S6. Anti-FGL2 treatment promotes DC-mediated proliferation of T cells in s.c. transplanted hepatomas in BALB/c mice. Figure S7. Akt phosphorylation in T cells is unaltered by sFgl2. (ZIP 2120 kb) [file 13046_2019_1326_MOESM1_ESM.zip › FigS7.tif]
